# Successful Outcome of Autologous Stem Cell Transplantation for Relapsed or Refractory Germ Cell Tumors

**Published:** 2018-07-01

**Authors:** Seied Aasadollah Mousavi, Negin Abedinzadeh, Leila Taj, Amir Kasaeian, Kamran Alimoghaddam, Mohammad Vaezi, Mohammad Zokaasadi, Hosein Kamranzadeh Fumani, Ardeshir Ghavamzadeh

**Affiliations:** Hematology, Oncology and Stem Cell Transplantation Research Center, Tehran University of Medical Sciences, Tehran, Iran

**Keywords:** Antineoplastic combined chemotherapy protocols, Stem cell transplantation, Neoplasms, Germ cell and embryonal, Survival analysis

## Abstract

**Background: **Treatment of choice for patients with refractory germ cell tumors (GCT) or recurrence after platinum containing chemotherapy regimens is not yet well recognized. This study is aimed to evaluate the role of high-dose chemotherapy (HDCT) followed by an autologous hematopoietic stem cell transplantation (ASCT) as the second-or third-line of salvage therapy in GCT patients.

**Materials and Methods:** Since 1997 to 2013, 13 GCT patients failing at least one salvage chemotherapy protocol were included in the study. The patients underwent chemotherapy, and then after a primary response the ASCT was performed. Survival analysis was done using Kaplan-Meier method.

**Results:** Eleven patients were male and 2 were female. All patients had gonadal tumors except one that had mediastinal GCT. Median follow-up time was 5.45±3.19 years. The estimated 5-year overall and disease-free survival rates were 84.00% and 69.23%, respectively. Five relapses after ASCT and 2 deaths occurred, and the cause of death was due to the relapse of primary disease in both cases. Transplant-related mortality (TRM) did not happen among the study participants.

**Conclusion:** our results showed acceptable outcomes for ASCT in refractory or relapsed GCT in terms of survival and treatment-related mortality. Larger prospective studies will be required to elucidate different aspects of such an interpretation.

## Introduction

Germ cell tumors (GCT) are a varied group of benign and malignant neoplasms derived from primordial germ cells. They occur in both gonadal and extra gonadal sites. GCT accounts for about 12 % of cancers in the adolescent group (15-19 years) ^1^. Therefore, GCTs are responsible for a great average number of life loss among adult malignancies. Anyhow, GCT is one of the most curable solid neoplasms by initiating chemotherapy even in the presence of metastatic disease ^2^ The International Germ Cell Cooperative Group (IGCCCG) classification is being used globally to guide first-line standard pharmacological treatment ^3^, which is usually consists of Cisplatin-based combination named Bleomycin, Etoposide and Cisplatin (BEP) or without Bleomycin (EP) ^4^. Although 40 to 80 % of patients may achieve curable remission which is a considerable high cure rate ^5^ depending on pretreatment clinical features, a significant proportion (10-20 %) will relapse and fail to achieve long-term disease- free survival ^6^.

For patients with primary resistant or relapsed disease after first-line treatment, there are second- and third-line treatments that offer potential for cure with lower chance of survival and greater risk of treatment-related morbidity compared to the first-line treatment ^7^. Optimal treatment at relapse after initial chemotherapy for GCT is poorly defined. Both conventional dose chemotherapy (CDCT) and high-dose chemotherapy with autologous stem cell transplantation (HDCT-ASCT) have been utilized ^8^. Because of uncertainty on the use of the best choice for first-line salvage therapy, CDCT versus HDCT-ASCT is being investigated in many randomized clinical trials, and impressive results have been reported with either approach. Most of the data indicate that cure rates for relapsed and refractory GCTs are only 25 % with conventional dose chemotherapy [9], HDCT-ASCT increases response rates and survival but with significant morbidity and treatment-related mortality between 3% and 21% ^10^^.^ However, reports comparing HDCT-ASCT versus CDCT are limited. Therefore, no standard treatment for refractory or relapsed GCT has been established to date ^8^.

Randomized trials comparing HDCT-ASCT versus chemotherapy as the first-line salvage therapy in GCT has not shown superior efficacy for transplant in terms of complete and partial responses to treatment ^11^^.^ While evidence about preferred choices of salvage therapy in the second-line or more is not available, this study was designed in order to evaluate the role of HDCT-ASCT after failure or one or more lines of salvage chemotherapy. 

## MATERIAL AND METHODS

Since January 1997 to February 2013, 13 patients with a confirmed diagnosis of GCT were included in this study. All diagnoses were based on the pathologic findings of the patients’ specimens after surgery and both gonadal and extra-gonadal forms were included. Inclusion criteria were defined as follows: relapsed disease after initial therapy or primary refractory disease and age ≥ 18 at the time of diagnosis. Patients diagnosed with non-germ-cell gonadal tumors were excluded from the study. Neutrophil and platelet engraftment were defined as neutrophil and platelet counts of more than 500/microliter and 20000/microliter, respectively. All patients received at least one platinum containing chemotherapy regimen and failed to achieve or sustain a durable remission. All autologous transplants were performed using peripheral blood stem cells and conditioning regimen included etoposide (500 mg/m^2^ for 3 days), carboplatin (600 mg/m^2^ for 2 days) and cyclophosphamide (1.6 mg/m^2^ for three days). In some cases, cyclophosphamide was replaced by ifosfamide. ASCT was performed when patients receiving chemotherapy showed a primary response to treatment. Primary response was defined as the presence of an overall response based on response evaluation criteria in solid tumors (RECIST). After the initiation of salvage chemotherapy, the patients’ response to therapy was evaluated at the end of each 2 courses, and when an overall response was observed, they were allowed to receive the ASCT. After confirmation of overall response, the following procedures were performed in a timely manner: stem cell collection, HDCT and ASCT. Informed consent was obtained from all patients and the data were kept confidential. The study protocol was approved by the institutional ethics committee.

All patients were followed up until death, relapse or the end of the expected follow-up time which was December 31^st^, 2016. The data were retrospectively analyzed and the Kaplan-Meier estimate was used to compute the overall and disease -free survival rates. Differences between groups analyzed by log-rank test and p-values of less than 0.05 were considered significant. The median follow-up time was calculated based on a reverse Kaplan-Meier method. All analyses were done in Stata, version 11.2 and R version 3.3.2 for windows.

## Results

 Eleven patients (84.62%) were male and the remaining two (15.38%) were female. The mean age at the time of diagnosis and transplant was 25.02±11.77 and 27.05±11.91 years, respectively. Basic characteristics of the patients are summarized in Table 1.

**Table 1:** Basic characteristics of patients

***Covariate***	***Category***	***Description***
*Sex*	Male	84.6% (n=11)
	Female	15.4%(n=2)
*Tumor pathology*	Seminoma	7.7%(n=1)
	Non-seminoma	61.5%(n=8)
	Mixed	30.8%(n=4)
*Primary tumor site*	Testis	76.9%(n=10)
	Ovary	15.4%(n=2)
	Mediastinum	7.7%(n=1)
*Presence of distant * *metastasis*	Yes	46.2%(n=6)
	No	53.8%(n=7)
*Primary chemotherapy*	BEP*	69.2%(n=9)
	Other platinum containing regimens	30.8%(n=4)
*First salvage * *chemotherapy*	TIP*	23.1% (n=3)
	VIP*	61.5% (n=8)
	Other	15.4% (n=2)
*Second salvage * *chemotherapy*	Yes	53.8% (n=7)
	No	46.2% (n=6)
*Radiotherapy*	Yes	15.4%(n=2)
	No	84.6%(n=11)
*Response to * *primary therapy*	Primary refractory	38.5%(n=5)
	Relapse	61.5%(n=8)
*Relapse after HSCT*	Yes	38.5%(n=5)
	No	61.5%(n=8)
*Survival status*	Alive	84.6%(n=11)
	Dead	15.4%(n=2)

*BEP: Bleomycin, Etoposide and Cisplatin. *VIP: Etoposide, Ifosfamide and Cisplatin.*TIP: Paclitaxel, Ifosfamide and Cisplatin.

**Fig 1:**
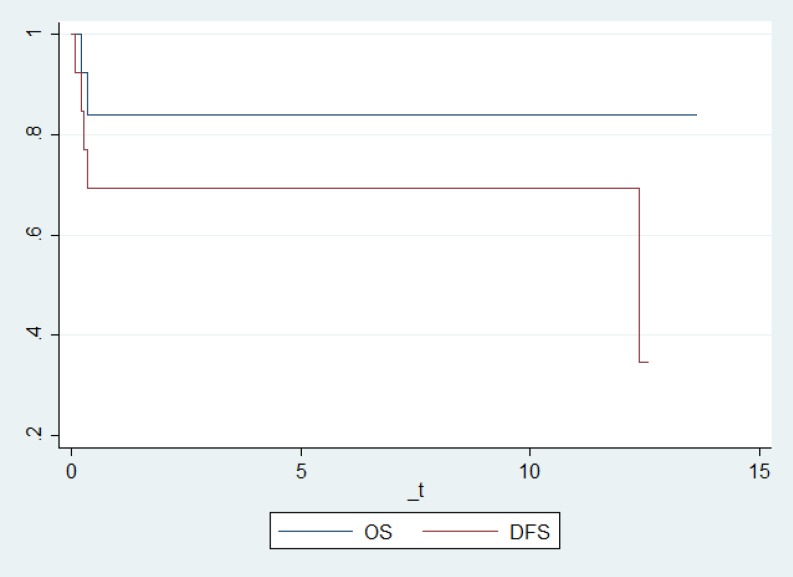
Overall and disease-free survival

The median time to engraftment was 15 days for neutrophil and 18 days for platelet lineages. At the end of the follow-up time, relapse was seen in 5 patients and death occurred in two cases. By a median follow- up time of 5.45±3.19 years, the estimated 5-year overall and disease-free survival rates were 84.00% and 69.23%, respectively (Figure 1).

**Fig 2:**
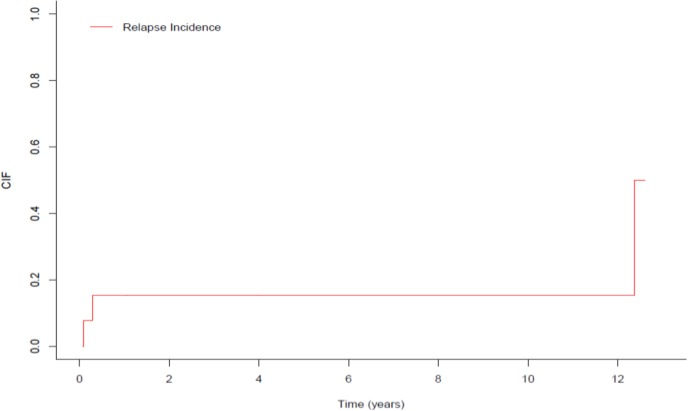
Cumulative incidence of relapse, CIF: cumulative incidence function

Cumulative five-and ten-year relapse incidence was 30.77% (95% CI: 12.82% - 62.66%) (Figure 2).

Transplant-related mortality (TRM) was not observed in our study. One patient developed renal failure during follow-up time and underwent kidney transplantation. The most common treatment complication was febrile neutropenia which was occurred in 4 patients (30.77%). The other toxicities are demonstrated in Table 2.

**Table 2:** Treatment-related toxicities

***Toxicity***	***Febrile *** ***neutropenia***	***Diarrhea***	***Mucositis***	***None***
*Frequency*	30.77% (n=4)	7.69% (n=1)	23.08% (n=3)	38.46% (n=5)

The median DFS time was 7.87±3.65 years. Using a log-rank analysis, no significant difference was observed between the OS and DFS of the patients when stratified for response to primary chemotherapy, using HCT-ASCT as the second or third salvage, the presence of distant metastasis, type of salvage regimens, tumor pathology, site and complications of treatment.

## Discussion

 High-dose chemotherapy followed by autologous hematopoietic stem cell transplantation is a potentially curative treatment of relapsed and refractory GCT, with long term post-transplantation survival as high as 70 % ^12^. It appears to provide a chance of cure for patients deemed otherwise incurable by conventional chemotherapy. In this study, the estimated 5-year overall survival rate (84%) showed better prognosis than other comparable published reviews^13^^,^^11^. Nabil Adra et al., conducted a retrospective analysis of 364 patients with relapsed or refractory GCTs undergoing HDCT-ASCT. With a median follow-up of 3.3 years, the 2-year overall survival was 66% ^14^. An alternative explanation for excellent overall survival rate in our study could be due to the smaller sample size in comparison to the studies mentioned above. Another reason could be the use of three-agent preparative regimen in this study in comparison to the regimen involving carboplatin and etoposide in the Indiana university experience.

 A retrospective study evaluated outcomes of a multi-institutional database of 1594 relapsed or refractory GCT patients treated with different salvage chemotherapy regimens and reported that when HDCT-ASCT used as the first line salvage treatment, the risk of disease progression decreased about 56% in comparison with standard dose chemotherapy. Statistically, this means a significant improvement in overall survival with HDCT-ASCT^15^. In our study, all participants received HDCT-ASCT as second, third or later line of salvage therapy and choosing the initial salvage chemotherapy in relapsed or refractory GCT patients remains controversial. In this regard, determining which patient can be a suitable candidate to receive salvage standard dose chemotherapy or HDCT-ASCT is one of the challenges that should be encountered in upcoming studies. Despite of receiving HDCT-ASCT other than first-line treatment, survival outcome in our study is superior to those studies giving HDCT-ASCT as first- line salvage therapy ^14^^,^^16^.

By resulting in no transplant-mortality (TRM) in our study, we achieved a comparable outcome to those studies showing 1.8 to 7% TRM ^10^^,^^17^^,^^2^^,^^6^^,^^11^ and a considerably better result in comparison to studies with higher rates like 21% in the study of Nichols CR et al^18^.

Our study showed the estimated 5-year disease-free survival rates of 69.23% and median DFS time of 7.87±3.65 years, while some other studies reported lower 5-year disease-free survival rates and shorter median DFS time ^10^^,^^17^.

Chemotherapy having Cisplatin in its combination resulted in 70% cure rate in newly diagnosed metastatic GCTs, reported by evaluating more than 5000 patients^10^. For patients who progress or relapse after first-line treatment, however, the issue of prognostic and survival factors is far more complex. In previous studies, variables such as the response to primary chemotherapy, presence of distant metastasis, type of salvage regimens, tumor pathology and site were considered as prognostic factors^8^, however, in our study these variables did not show statistically significant differences in order to be accounted as prognostic factors due to the small sample size.

In a study by Pico JL et al., the effect of HDCT-ASCT as the first line of salvage in GCT patients was evaluated and revealed no significant survival advantage for stem cell transplant. Moreover, TRM was higher in the arm undergoing ASCT compared to the chemotherapy-only arm^11^. Our study was conducted in patients failing primary platinum-containing therapy plus at least one salvage chemotherapy regimen. But, still the ASCT leads to favorable results.

## COCLUSION

 In this study, we have shown promising outcomes of high-dose chemotherapy, followed by autologous stem cell transplantation for GCT. It should be considered that even when it was used as second, third or later lines of salvage therapy, HDCT-ASCT could lead to significant OS and DFS improvement for refractory and relapsed germ cell tumors. Further prospective studies with larger sample size are needed to evaluate other variables such as beta HCG and alpha fetoprotein as prognostic factors. Also, the assessment of giving HDCT-ASCT as first-line salvage therapy can provide better perspective in order to offer more beneficial treatments.
